# Hepatoprotective effects of diosmin: a narrative review

**DOI:** 10.1007/s00210-024-03297-z

**Published:** 2024-08-21

**Authors:** Emad H. M. Hassanein, Hanan S. Althagafy, Mohammad A. Baraka, Haitham Amin

**Affiliations:** 1 Department of Pharmacology and Toxicology, Faculty of Pharmacy, Al-Azhar University, Assiut Branch, Assiut, 71524 Egypt; 2 Department of Biochemistry, Faculty of Science, University of Jeddah, Jeddah, Saudi Arabia; 3 Faculty of Pharmacy, Al-Azhar University, Assiut Branch, Assiut, 71524 Egypt; 4 Department of Pharmaceutics and Pharmaceutical Technology, Faculty of Pharmacy, Al-Azhar University, Assiut, 71524 Egypt

**Keywords:** Diosmin, Liver diseases, Hepatoprotective, Antioxidant, Anti-inflammatory

## Abstract

Liver diseases represent a formidable global health threat. Hesperidin, a flavonoid found in citrus fruits, is the source of diosmin (DS). The in vivo and in vitro investigations of the pharmacological effects of DS reveal that it exhibits tremendous beneficial effects, such as fighting against inflammation, oxidative stress, and fibrosis. These effects have been noticed in various disease models, emphasizing the potential therapeutic value of DS in tackling diverse pathological conditions. Interestingly, DS has promising liver-defense capabilities against a range of hepatic illnesses, such as radiation-induced hepatic injury, liver ischemia/reperfusion injury, alcoholic hepatic disease, nonalcoholic fatty liver disease (NAFLD), and hepatocellular carcinoma (HCC). Furthermore, DS demonstrates potential hepatoprotective effects against environmental toxins, such as heavy metals. DS activates PPAR-γ and Nrf2, leading to antioxidant effects that reduce oxidative stress. Moreover, DS suppresses NF-κB, NLRP3, MAPK activities, and cytokine production (TNF-α and IL-1β), resulting in inflammation suppression. These anti-inflammatory effects are attributed to the activation of PPAR-γ and Nrf2, which are NF-κB inhibitors. This review aims to comprehensively discuss the hepatoprotective capacity of DS, elucidating the underlying mechanisms and identifying several research avenues that warrant further exploration to ascertain the prospective clinical advantages of DS intake as a viable strategy for the treatment of hepatic illnesses.

**Graphical Abstract:**

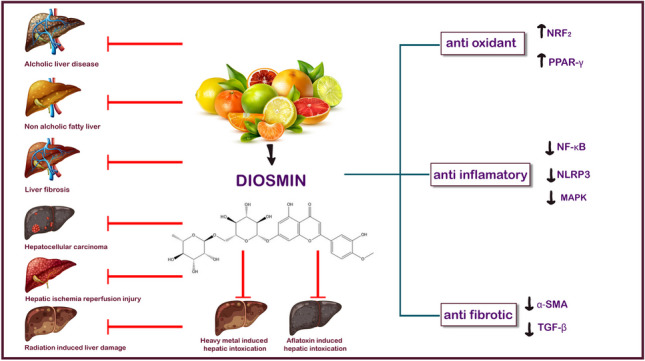

## Introduction

### Hepatic diseases as a global challenge

The liver, the largest internal organ in the body, is situated in the abdominal cavity on the right side, beneath the diaphragm. The liver has a unique blood supply that empowers it to perform a number of vital functions. These tasks include the following: firstly, toxins’ elimination; secondly, plasma glucose regulation; thirdly, blood homeostasis; and lastly, several other crucial functions (Ozougwu and Biosciences 2017). It is continually subjected to a tremendous number of xenobiotics, environmental pollutants, and other toxins. This exposure is both continuous and varied (Mohi-Ud-Din et al. 2019). Globally, both acute and chronic liver diseases are common (Beste et al. 2015; Castellví et al. 2017; Mokdad et al. 2014). Approximately 2 million fatalities worldwide are attributed to liver disorders each year. Every year, one million people pass away from cirrhosis-related problems, and the other million pass away from HCC and viral hepatitis (Asrani et al. 2013; Mokdad et al. 2014). Liver illnesses accounted for 2.2% of all fatalities worldwide in 2016, ranking as the 11th highest cause of mortality and the 15th greatest source of morbidity globally. Nearly 1.32 million people lost their lives to chronic liver disease in 2017. Out of these 1.32 million deaths, two-thirds were male, and the remaining one-third were female (Cheemerla and Balakrishnan 2021). Hepatotoxicity may result from toxins impairing the liver’s antioxidant defense mechanisms. The crucial causes of hepatic damage are free radicals from oxidative stress, inflammatory conditions, degeneration, and necrotic death of hepatic cells. Liver damage can result from a number of conditions, including viral infections, drug-induced liver injury (DILI), NAFLD, alcoholic liver disease (ALD), and hepatic ischemia‒reperfusion (HIR). These are the most classic scenarios of liver damage. HCC and liver cirrhosis can arise from liver fibrosis, which is a consequence of chronic liver injury (Asrani et al. 2019; Shojaie et al. 2020).

According to estimates from the WHO, chronic diseases account for 59% of deaths and 46% of illnesses worldwide. Every year, 35 million people worldwide pass away from chronic illnesses, and this figure is spiraling upward (Bengmark and Nutrition 2006). Healthcare costs have steadily risen in recent decades and this trend is expected to continue and even accelerate. According to the United Kingdom’s Office for National Statistics, hepatic disease is currently the fifth leading cause of mortality behind cardiac disease, stroke, pulmonary disease, and malignancy (Johnson et al. 2005). Consequently, there is an urgent need to prevent and treat hepatic damage. Addressing such severe health problems demands more effort and resources to counteract the adverse impacts on liver health.

### Diosmin

#### Source

Flavonoids, a family of phytochemicals, are well known for their unique clinical qualities, such as the suppression of inflammation and free radical generation. Many studies have shown that flavonoids can alleviate both inflammation and oxidative stress owing to their unique chemical features (Rice-Evans et al. 1996). Flavonoids work as effective free radical scavengers and powerful metal chelators. Studies have also revealed that flavonoids are much safer than other phytochemicals (Middleton et al. 2000). Flavonoids are polyphenolic compounds that possess a benzo-γ-pyrone basic nucleus that contains numerous hydroxyl groups (Mahomoodally et al. 2005). Approximately 4000 different flavonoid structures are predominantly dispersed across the subclasses of flavonols: flavonols, flavanols, flavanones, isoflavones, and flavanonols. These flavonoids occur naturally in seeds, herbs, fruits, vegetables, tea, spices, and whole grains, all of which constitute common components of a normal human diet (Galluzzo et al. 2008; Hughes et al. 2008).

Diosmin (DS), also known as diosmetin 7-O-rutinoside, is a flavonoid phytochemical obtained from citrus. Studies have revealed that DS have the capacity to minimize oxidative stress and inflammation owing to their intrinsic chemical characteristics (Ali et al. 2018a, b Bakr et al. 2020). Research on DS dates back to 1925, when it was initially isolated from a wort plant (Bogucka–Kocka et al. 2013; Huang et al. 2011). Since then, DS has been employed as a natural therapeutic alternative for a variety of circulatory conditions, including leg ulcers, varicose veins, hemorrhoids, and related problems. Its utility as a natural therapy for various circulatory problems came from its initial discovery approximately a century ago (Bogucka–Kocka et al. 2013; Huwait and Mobashir 2022; Zheng et al. 2020). DS is a natural bioflavonoid that is obtained from the removal of hydrogen from hesperidin. Hesperidin is abundant in the peels of citrus fruits, as well as other medicinal plants, from which it is collected and subsequently transformed into DS (Campanero et al. 2010; Freag et al. 2013; Luigi Silvestro et al. 2013). An oral flavonoid medication, Daflon, is composed of 10% hesperidin and 90% diosmin and has phlebotonic and venoprotective capabilities (Lenkovic et al. 2012). Daflon is frequently used to treat blood vessel disorders such as varicose veins (Bush et al. 2017), chronic venous insufficiency (Maksimović et al. 2008), and piles (Shelygin et al. 2016). Furthermore, DS exhibits numerous advantages, such as combating inflammation, free radical generation, and fibrosis (Ahmed et al. 2016; Samar H Gerges et al. 2020; Jain et al. 2014). Several researchers have studied the possible preventive or curative advantages of DS in animal models of several illnesses, including colon ulceration (Shalkami et al. 2018), peptic ulcer (Arab et al. 2015), hyperglycemia (Srinivasan and Pari 2012), and several toxicities (Abdel-Daim et al. 2017).

#### Chemistry

DS has the molecular formula C_28_H_32_O_15_. Diosmetin7-neohesperidoside, 3′,5,7-trihydroxy-4′-methoxyflavone 7-rhamnoglucoside, 3′,5,7-trihydroxy-4′-methoxyflavone-7-rutinoside, and diosmetin 7-O-rutinoside are chemical names for DS. DS is a flavone glycoside, a flavonoid molecule with a sugar component connected to a three-ring flavonoid basic nucleus (Russo et al. 2018).

#### Pharmacokinetics

##### Absorption

When taken orally, DS is broken down by intestinal microbiota enzymes into its aglycone form, diosmetin, which is subsequently passively absorbed through the intestinal wall (Garner et al. 2002; Patel et al. 2013). Research has indicated that diosmetin is actively transported. This finding was verified by an investigation exploring the uptake of various flavanones and flavones into a Caco-2 cell monolayer. According to the study, diosmetin uptake was five times greater in the apical-to-basolateral direction than in the basolateral-to-apical direction, indicating the potential involvement of an active transport mechanism (Serra et al. 2008). Unfortunately, diosmetin serum levels after an oral dosage of DS have been reported to be low and fluctuating (Garner et al. 2002; Russo et al. 2018). Compared to non-micronized DS, the administration of micronized DS, which has a smaller particle diameter, to healthy individuals remarkably improved the intestinal absorption of diosmetin, as indicated by a notorious increase in its urinary excretion (Garner et al. 2002).

##### Distribution

When rats were orally administered diosmin to hesperidin (9:1), diosmetin had a longer half-life and a larger volume of distribution (V_d_) than the whole blood, revealing that diosmetin is widely uptaken into tissues (Bhattacharyya et al. 2019). Studies have demonstrated that diosmetin has a long half-life after delivery of DS to normal individuals (D Cova et al. 1992). Although the distribution of diosmetin in humans is unknown, research has shown that DS binds firmly to albumin (Barreca et al. 2013; Poór et al. 2018), suggesting that it may circulate widely throughout the body in a manner similar to findings from animal studies (Mrkalić et al. 2021). Likewise, another study indicated that DS can displace warfarin from the shoulder of plasma protein, minimizing the binding to almost 40% (Poór et al. 2018).

##### Metabolism

In 2019, a study evaluated the metabolism of DS and diosmetin in rats. This research detected 64 DS metabolites and 46 diosmetin metabolites in different body excretions, such as blood, urine, and feces. These metabolites included conjugates of glucuronide and sulfate, as well as demethylated, methylated, glycosylated, and hydroxylated forms. The findings showed that before being absorbed, DS is not only converted into diosmetin; diosmetin is further metabolized by intestinal flora or first-pass effects via the liver, which produces a variety of chemicals (Chen et al. 2019). Some enzymes, including CYP1A1, CYP1A2, and CYP1B1, assist in diosmetin demethylation. The production of luteolin, which has certain biological characteristics and exhibits some activity, is the result of this enzymatic activity (Androutsopoulos et al. 2009). Luteolin is glucuronidated to form glycoconjugates, which have their own pharmacological actions (Wang et al. 2017).

Diosmetin-7-O-glucoside, a powerful liver-protective molecule, was also discovered (Chen et al. 2019; Wang et al. 2016). In addition to the aforementioned enzymes, diosmetin is also metabolized via the CYP3A4 enzyme. The concentration of the parent molecule decreased after a 20-min incubation with CYP3A4. However, no significant decline in diosmetin levels was observed when diosmetin was incubated with different liver microsomal enzymes, including CYP3A5, CYP2B6, CYP2C9, CYP2C8, CYP2C19, CYP2E1, CYP2D6, or CYP2A6 (Androutsopoulos et al. 2009). During circulation throughout the body, diosmetin is metabolized to 3-O-glucuronide, which is then esterified to 3,7-O-diglucuronide, according to research on healthy subjects. Diosmetin 3-O-glucuronide was also discovered to be the major metabolite in the blood and urine (Silvestro et al. 2013). Studies have shown that when diosmetin is exposed to hepatic microsomal enzymes, it undergoes regioselective glucuronidation (Zeng et al. 2016). Several metabolites of DS, including luteolin and diosmetin-7-O-glucoside, have been shown to have biological activities (Wang et al. 2017, 2016).

##### Excretion

**Fig. 1:**
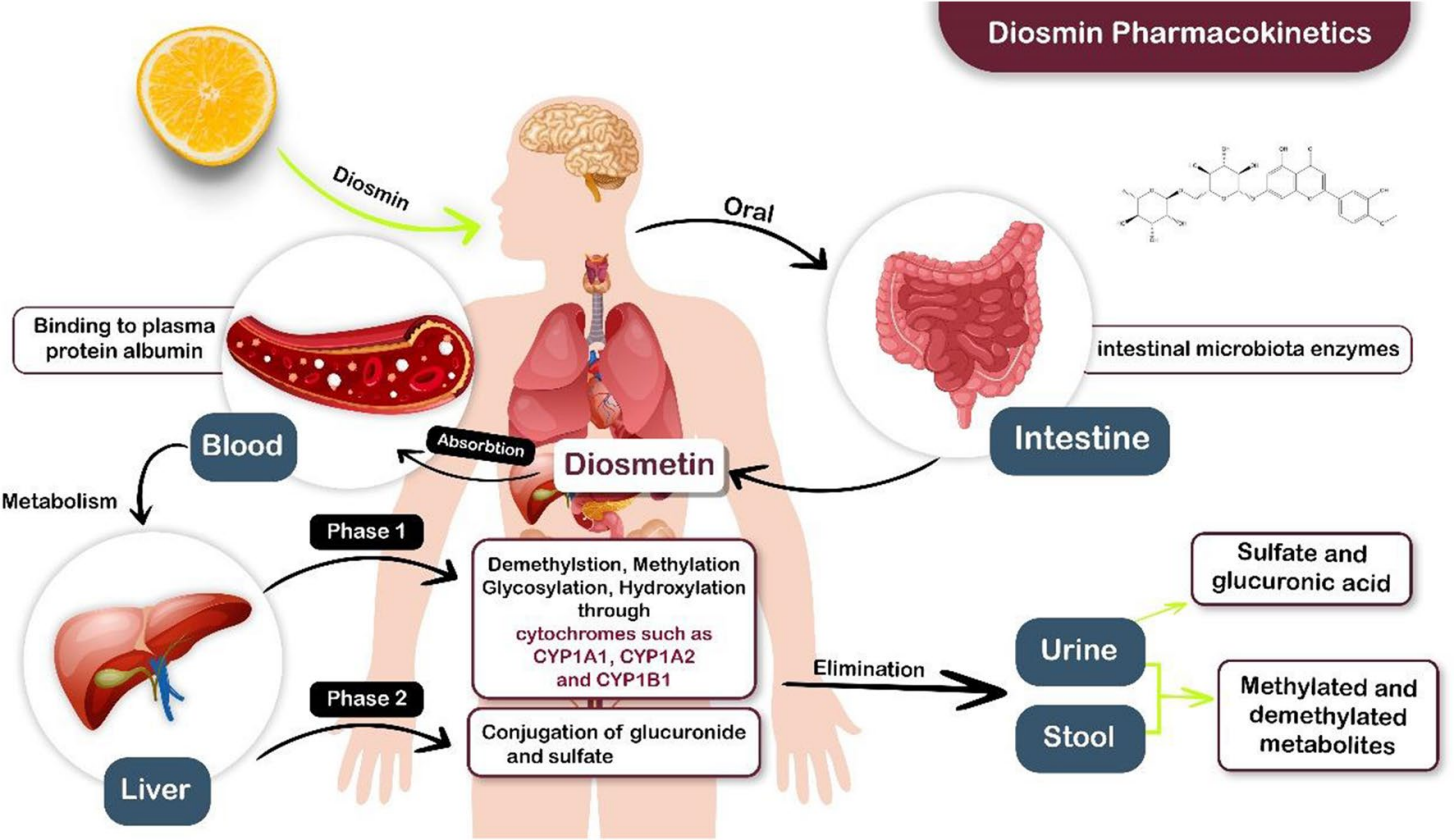
The pharmacokinetics of DS

Based on research performed by Chen et al. in 2019, urine acts as the predominant excretory channel for DS and diosmetin metabolites in rats. There were 42 diosmetin metabolites found in urine and 5 in stool, in addition to 51 diosmin metabolites found in urine and 17 in stool. Research has also confirmed the presence of demethylated and methylated forms of DS and diosmetin, together with their conjugates of sulfate and glucuronic acid, in both stool and urine samples (X. Chen et al. 2019). In 1992, research was conducted to assess the elimination of DS in individuals in good shape. The results demonstrated that the levels of DS and its principal metabolite, diosmetin, were entirely absent in the urine samples. This discovery indicated that diosmetin is entirely absorbed from the gut, as described earlier, and undergoes full metabolism and conjugation inside the circulation (D. Cova et al. 1992). Diosmetin glucuronic acid conjugates were discovered in human urine samples (D. Cova et al. 1992). Furthermore, glucuronides are the main conjugates found in urine, according to Silvestro et al. However, they also identified modest levels of sulfate conjugates (Silvestro et al. 2013). The pharmacokinetics of DS are illustrated in Fig. 1 and summarized in Table 1.

**Table 1:** Pharmacokinetics data of DS in both preclinical and clinical models

Pharmacokinetics	Preclinical or clinical models	Main findings	References
Absorption	Healthy volunteers	DS undergoes hydrolysis by intestinal flora, resulting in the formation of the aglycone diosmetin, which is subsequently absorbed through the intestinal wall The plasma concentration of diosmetin after oral administration of DS is low and showing fluctuations	(Garner et al. 2002)
	Rats	The bioavailability of diosmetin has been reported to be approximately 3.6% After orally administering a combination of DS and hesperidin in a ratio of 9:1	(Bhattacharyya et al. 2019)
	Healthy volunteers	Pharmacokinetic parameters for diosmetin demonstrate an area under the curve (AUC) of 298.4 ± 163.7 ng·h/ml, a maximum plasma concentration (C_max_) of 50.3 ± 22.6 ng/ml, and a time to reach maximum concentration (T_max_) of 2.2 ± 2.9 h. In contrast, the micronization process significantly alters these parameters. For micronized DS, the AUC of diosmetin is considerably lower at 31.9 ± 100.4 ng·h/ml, and the C_max_ is reduced to 2.4 ± 1.9 ng/ml Micronized DS, in combination with a buffering agent (μSmin® Plus), increased the plasma concentrations of diosmetin and enhanced bioavailability compared to micronized DS alone	(Russo et al. 2018)
Distribution	Rats	The volume of distribution of diosmetin surpasses the total blood volume of the rat (0.054 L/kg), and it demonstrates a prolonged half-life ranging from 26 to 43 h Diosmetin exhibits extensive distribution into various tissues	(Bhattacharyya et al. 2019)
	Human serum albumin	The presence of DS decreases the binding affinity of olanzapine to human serum albumin through competition, with a binding constant of 2.77 × 10^−5^ M^−1^, compared to 7.1 × 10^−5^ M^−1^ in the absence of DS DS binds to human serum albumin and can competitively interact with medications for binding sites	(Barreca et al. 2013; Mrkalić et al. 2021)
Metabolism	Rats	A total of 46 diosmetin and 64 DS metabolites were detected in the blood, urine, and feces Various metabolites of DS and diosmetin have been identified, including methylated, demethylated, glycosylated, and hydroxylated forms, as well as glucuronide and sulfate conjugates	(Chen et al. 2019)
	Healthy volunteers	In systemic circulation, diosmetin undergoes esterification to form its 3-O-glucuronide, which is subsequently esterified further to produce the 3,7-O-diglucuronide Diosmetin 3-O-glucuronide was identified as the predominant metabolite in both plasma and urine	(Luigi Silvestro et al. 2013)
Excretion	Rats	Methylated and demethylated metabolites of both DS and diosmetin have been identified in the urine and feces The urinary route is the primary pathway for excretion	(Chen et al. 2019)
	Healthy volunteers	Minimal metabolites are excreted in the urine, primarily as conjugates of glucuronic acid Complete lack of excretion in the urine of DS and its aglycone diosmetin; minimal metabolites are excreted in the urine, primarily as conjugates of glucuronic acid. A metabolism resembling that of other flavonoids is confirmed by the presence of breakdown products including alkyl-phenolic acids	(Cova et al. 1992)
	Healthy volunteers	The main conjugates found in the urine are glucuronides, with sulfates present in lower amounts	(Silvestro et al. 2013)

#### Extraction of DS

Diosmin is a greyish-yellow or pale-yellow hygroscopic powder (Bogucka–Kocka et al. 2013). DS, hesperidin, and other flavonoids were extracted from citrus peels, with the study assessing the impact of water content (0% and 75%), extraction time (30 and 90 min), and peel-to-water ratio. Peels from Tahiti lime, sweet orange, and Oneco tangerine were used. The highest yield was obtained from dry peels with a 30-min extraction, while the lowest yield came from wet peels with the same extraction time. Yield from wet peels increased with longer extraction times. Overall, dry peels yielded higher flavonoid content, and extraction time did not impact the phenolic content (Londoño-Londoño et al. 2010). DS, in contrast to most flavonoid compounds, exhibits very poor solubility in both polar and nonpolar protic solvents but is readily soluble in certain aprotic solvents, such as DMSO. This feature assists in the easy separation of DS from other accompanying flavonoids. This approach follows the method suggested by Ivashev et al. (1995). The plant material (*Hyssopus officinalis* L., Lamiaceae) was defatted using chloroform and then treated with aqueous ethanol or water, followed by 96% ethanol. DS was subsequently extracted from the plant material using dimethyl sulfoxide (DMSO). The combined extracts were then added to a ten-fold amount of water forming a crystalline precipitate of DS within 48 h. For further purification, recrystallization from DMSO-water and DMSO-methanol mixtures, hand in hand with ethanol washing, was recommended to get rid of accompanying compounds (Ivashev et al. 1995).

#### Biological activities of DS

Several studies have been conducted in vivo, in vitro, and in clinical settings to assess the potential pharmacological effects of DS. This review demonstrated that DS possesses many upsides, including anti-inflammatory and antioxidant activities. DS could scavenge free radicals, thereby lowering oxidative stress, and possess antidiabetic (Hsu et al. 2017), antihyperlipidemic (Firdous et al. 2021), and anticancer (Perumal et al. 2018) effects. DS could achieve a resounding success as an effective and safe therapy for a broad variety of illnesses. Notably, it can inhibit multiple metabolic enzymes, driving clinical studies to investigate its therapeutic effectiveness and safety under diverse conditions while addressing possible interactions. As a venoprotective medication, DS exerts its effects on blood vessels via numerous pathways, resulting in enhanced blood flow. DS improves venous tone and flexibility, microcirculation, and lymphatic drainage. Due to these features, DS is frequently employed to boost vascular wellness in persons with chronic venous illness, besides improving their overall quality of life (QoL) (Bozdağ and Eraslan 2020; Carballo-Villalobos et al. 2016; Gerges et al. 2022; Waring et al. 2015).

#### Safety

Numerous clinical investigations have provided evidence of the useful effects of DS. Moreover, toxicological examinations have demonstrated a satisfactory safety profile for DS. As a consequence, DS offers the potential to be a successful and safe therapy for different illnesses. Furthermore, both clinical and experimental toxicological investigations have carefully studied the toxicity and possible adverse effects of DS, continuously indicating a satisfactory long-term safety profile (Meyer 1994; Söylemez et al. 2012). In research comprising 327 volunteers who satisfied the requirements, micronized DS was provided at a daily dosage of either 1000 mg or 2000 mg for four months. The participants’ parameters were assessed at baseline, after 2 months, and after 4 months. The outcomes of the research revealed that no substantial adverse events were recorded in either of the study groups (Staniewska 2016). In another study, 60 volunteers who satisfied the requirements were separated into two groups. One group received conventional medicine, whereas the other group received conventional medication coupled with a mixture of natural items, including DS. Throughout the trial, no negative impacts were observed in either group. Furthermore, clinical and laboratory studies have not revealed any signs of systemic toxicity arising from the combination of natural products containing DS (Cacchio et al. 2019).

### Hepatoprotective effects of DS

#### Nonalcoholic fatty liver disease (NAFLD)

NAFLD is a condition characterized by the accumulation of extra fat in the liver, referred to as steatosis, in the absence of heavy alcohol consumption. In 2017, NAFLD emerged as a global epidemic with a considerable global incidence (Younossi et al. 2018). NAFLD may manifest in two forms: simple fatty liver or steatosis, characterized by fat buildup in liver cells, or nonalcoholic steatohepatitis (NASH) (Cohen et al. 2011). Furthermore, NASH has the potential to progress to HCC (Anstee et al. 2019). The original etiology of NASH is complicated and includes multiple variables, for instance, insulin resistance (IR), overweight status, and adipocyte anomalies (Manne et al. 2018). Lipid-rich diets enhance the transfer of large amounts of free fatty acids (FFAs) to hepatic cells via the portal vein (Fielding 2011). Furthermore, peripheral insulin resistance (IR) and being overweight result in enhanced lipolysis, leading to the formation of extra FFAs. These FFAs are subsequently transferred to the liver (Fabbrini et al. 2010). Hepatic IR reduces glycogenesis while increasing gluconeogenesis, resulting in elevated glucose levels and concomitant hyperinsulinemia (Sanders and Griffin 2016). As a consequence, hyperinsulinemia raises hepatic de novo lipid synthesis, aggravates steatosis, and worsens hepatic IR (Saponaro et al. 2015; Williams et al. 2013). Lipotoxicity also activates Kupffer cells, boosting the production of TNF-α, IL-6, reactive oxygen species (ROS), and TGF-β (Seki et al. 2007). Furthermore, dysfunction of adipose tissue leads to an increase in inflammatory cytokines and a decrease in the production of adiponectin (Rotter et al. 2003). The stimulation of hepatic stellate cells (HSCs) by inflammatory cytokines, TGF-β, and ROS prolongs hepatic damage, eventually leading to fibrosis (Marra & Tacke 2014). The care of patients with NASH generally relies on limiting disease development by managing its risk factors, with a special emphasis on reducing IR and obesity (Puri 2017). Frequently utilized methods include weight loss (Promrat et al. 2010) and the use of medications that act as insulin sensitizers, hypocholesterolemic agents, and antioxidants (Abd El-Kader and El-Den Ashmawy 2015).

The underlying processes of DS were investigated in a study conducted on a rat model with a high-fat diet and streptozotocin-triggered NASH. The data demonstrated that DS therapy had various favorable effects. It considerably improved the histopathological NASH results; minimized the levels of TNF-α, IL-6, and malondialdehyde (MDA); boosted glucose and fat metabolism; and decreased liver TGF-β, α-SMA, and collagen content compared with those in untreated rats (Gerges et al. 2020).

#### Alcoholic liver disease (ALD)

ALD has become a serious worldwide health problem in recent decades. Over time, ethanol has gained a damning reputation as an addictive substance (Guo and Ren 2010). Drinking alcohol can cause liver damage and a number of alcoholic liver conditions, such as cirrhosis and steatohepatitis (Odriozola et al. 2023). Within the liver, ethanol undergoes conversion into a horrendous poisonous metabolite called acetaldehyde. This acetaldehyde subsequently engages with cell macromolecules such as proteins and lipids, leading to the breakdown of membrane phospholipids and changes in the activity of enzymes (Niemelä 1999). The metabolism of ethanol involves three major enzyme systems (Dunn and Shah 2016): alcohol dehydrogenase (ALDH), CYP450 2E1, and catalase. ALDH produces acetaldehyde from ethanol while simultaneously reducing NADP to NADH, which drives up xanthine oxidase function and contributes to elevated superoxide generation (Lieber 2005). The activation of CYP450 2E1 is part of the microsomal alcohol oxidation mechanism (Lieber 2004).

The reduction of O_2_ to H_2_O_2_ occurs as a result of the oxidation of ethanol and nicotinamide adenine dinucleotide phosphate (NADPH). In a catalase-catalyzed reaction, the oxidation of ethanol to acetaldehyde results in the fast breakdown of H_2_O_2_ (Bradford et al. 1993). Free radical production linked to ethanol metabolism causes oxidative damage (Cederbaum 2001; Cederbaum et al. 2009). Reactive nitrogen intermediates (RNIs) and ROS production are two hallmarks of oxidative stress that significantly influence the pathophysiology of ethanol-triggered hepatic injury. The balance between oxidation inducers and oxidation suppressors within tissues is altered by this process (Arteel 2003). The oxidative metabolism of ethanol produces acetaldehyde, which then causes the peroxidative degradation of membrane phospholipids, resulting in harm to cells. Furthermore, acetaldehyde can engage with a wide range of cell proteins, such as microtubules, proteins, and microsomal enzymes, thereby impairing their normal function (Rintala et al. 2000). Acetaldehyde has been suggested to be a key factor in the development of ALD. Additionally, acetaldehyde can interact with DNA to form cancerous DNA adducts, including N2-ethyl-2′-deoxyguanosine (Brooks and Theruvathu 2005). The toxicity of ethanol stimulates NF-kB, which ultimately results in producing inflammatory mediators, such as TNF-α, IL-6, and IL-12 (Iimuro et al. 1997), as well as chemokines, lipid mediators, iNOS, and COX-2, resulting in the production of ROS and RNI, causing liver damage (Barnes and Karin 1997; Nanji et al. 1999).

With DS treatment, there were notable reductions in both relative liver weights and weight loss triggered by ethanol. ADH and CYP 2E1 aid in the oxidation of ethanol, which results in the generation of NADH, which is thought to be a crucial element in the pathophysiology of alcoholic hepatic injury (Zima et al. 2001). The levels of TNF-α, NF-kB, iNOS, and COX-2, which are among the inflammatory and necrosis markers that are enhanced in rats treated with ethanol alone, are the main indicators of liver injury caused by continuous alcohol intake. However, treatment with DS reduced the levels of these skyrocketing inflammatory cytokines as well as the levels of signs of necrotic cell death. DS has shown the potential to inhibit the generation of ethanol-induced ROS and inflammatory mediators, consequently relieving ethanol-induced oxidative stress. Alcohol intake can lead to liver injury by producing free radicals, changing redox equilibrium, damaging mitochondria, peroxidizing membrane phospholipids, and activating both TNF-α and NF-kB. DS mitigates alcoholic liver damage by modifying ethanol metabolism, attenuating oxidative stress, and reducing inflammation (Tahir et al. 2013a, b).

#### Hepatic fibrosis

One of the leading causes of morbidity and mortality is hepatic fibrosis (Sánchez-Valle et al. 2012). Liver fibrosis emerges from the excessive buildup of ECM proteins within the liver, largely generated through activating HSCs. When subjected to proinflammatory and profibrogenic triggers, inactive HSCs undergo differentiation into myofibroblast-like cells that express α-smooth muscle actin (α-SMA). This mechanism eventually leads to the development of hepatic fibrosis (Liu et al. 2015; Tu et al. 2014). Wnt/β-catenin signaling is essential for several biological processes, including organogenesis, tissue homeostasis, and the incidence of certain illnesses. It is necessary for sustaining critical functioning in these processes (Clevers 2006). Prolonged stimulation of Wnt/β-catenin signaling activates HSCs and promotes their proliferation and buildup of the extracellular matrix (ECM) (Kordes et al. 2008).

MicroRNAs (miRNAs) are small RNA molecules with between 20 and 22 nucleotides. They serve a key function in regulating gene expression by either limiting protein translation or increasing the breakdown of mRNA molecules (Croce and Calin 2005). MiRNAs have the potential to either stimulate or inhibit HSCs, indicating that they operate as mediators of HSC activity in liver fibrosis (Yu et al. 2016). In recent investigations, the expression of miR-17-5p, which is a member of the miR-17–92 cluster, is elevated in several cancers, including HCC. It has been identified as an oncogenic miRNA, indicating that it promotes tumor development and progression (Shan et al. 2013). Recent investigations have shown that the overproduction of miR-17-5p leads to the activation and proliferation of HSCs. This shows that miR-17-5p could enhance HSC activity and lead to liver fibrosis (Yu et al. 2016).

Research on rats with hepatic fibrosis revealed that liver fibrosis caused by radiation may arise via disruption of the cell membranes of liver cells. This damage enhances membrane permeability, enabling cytoplasmic enzymes to leak out of the cells. As a consequence, there is an increase in serum aminotransferase activity (Gaur and Bhatia 2009). Hasan et al. hypothesized that the fibrosis-inhibiting effects of DS are mediated via the promotion of peroxisome proliferator-activated receptor-gamma (PPAR-γ) production and interaction with miR-17-5p, which triggers the Wnt-β-catenin pathway. The results demonstrated that DS therapy successfully suppressed pro-oxidants, stimulated antioxidants, relieved the liver inflammatory response, repressed fibrosis-inducing cytokines, and boosted PPAR-γ levels. Moreover, DS therapy inhibited Wnt-β-catenin signaling caused via the IRR (Hasan et al. 2017).

Moreover, Ali et al. reported that DS relieves fibrosis caused by bile duct ligation (BDL). This includes reductions in oxidative damage, fibrosis progression, and abnormal hepatic functions. The study also revealed that DS influenced the upregulation of nuclear factor-kappa B (NF-κB)-p65, p38-mitogen-activated protein kinases (p38MAPK), Kelch-like ECH-associated protein 1 (Keap-1), and inducible nitric oxide synthase (iNOS) while downregulating nuclear factor erythroid 2-related factor 2 (Nrf-2), cytoglobin, and endothelial NOS levels. These data imply that DS fights against cholestasis and hepatic diseases via various signals, including Keap-1/Nrf-2 and p38-MAPK/NF-κB/iNOS (Ali et al. 2018a, b). Similar studies have shown that the combination of DS and PTX may successfully alleviate liver dysfunction (Ali et al. 2018a, b). This synergistic effect is hypothesized to occur via the modulation of Keap-1/Nrf-2/glutathione (GSH) as well as NF-κB-p65/p38-MAPK signaling.

#### Hepatocellular carcinoma (HCC)

HCC is the most common type of primary hepatic cancer, accounting for almost one million fatalities per year. It is the fourth leading cause of fatalities due to cancer worldwide (Balogh et al. 2016). Chronic hepatitis B and C virus infection, susceptibility to aflatoxin exposure, smoking, and NAFLD associated with obesity are all major risk factors for HCC (Zheng et al. 2019). Preventive chemotherapy is a regularly adopted treatment for liver malignancies. The excessive generation of ROS causes oxidative metabolism of diethyl nitrosamine (DEN), resulting in oxidative stress as well as changes in gene expression and intracellular signaling pathways. Diethyl nitrosamine is commonly regarded as a highly carcinogenic chemical related to hepatocellular disorders (Pandey et al. 2018).

DS has been shown to efficiently reduce HCC induced by DEN. It helps stabilize fluctuating levels of enzymes, including AST, ALT, ALP, LDH, and xenobiotic enzymes. Additionally, DS improves liver histopathology, leading to favorable changes in liver tissues and function (Tahir et al. 2013a, b). The inhibition of hepatic cancer by DS may be related to its capacity to increase the activity of protein phosphatase 2A (PP2A). PP2A is genetically inactivated in several kinds of cancer. These data show that pharmacological treatments focused on boosting PP2A activity may hold promise as feasible therapeutic options for hepatic cancer. In their work, Dung et al. studied the effect of DS on HA22T human HCC cells both in vivo and in vitro in a mouse xenograft model. HA22T cells received different doses of DS, and later research demonstrated considerable enhancement of apoptosis in the cells. Furthermore, treatment with DS resulted in a decrease in tumor size in a xenograft model (Dung et al. 2012).

#### Hepatic ischemia–reperfusion injury

Hepatic ischemia–reperfusion (HIR) damage can lead to hepatic dysfunction as well as failure following surgery, trauma, hypovolemic shock, and liver transplantation (Eltzschig and Eckle 2011). ROS and inflammatory cytokines generated by hepatocytes and innate immune system cells play important roles in reperfusion damage and can lead to inflammation, and, eventually, liver failure (Jochmans et al. 2017; Monga 2018). Numerous studies have linked HIR damage to the activation of the Nod-like receptor protein-3 (NLRP3) inflammasome (Guo et al. 2016; Huang et al. 2013). Conversely, Nrf2, a vital modulator of cell defense, is central for preserving cellular redox homeostasis and is involved in the defense against HIR (Hassanein et al. 2021; Kamel et al. 2020; Mahmoud et al. 2019; Yamamoto et al. 2018).

The work by Tanrikulu et al. evaluated the protective benefits of DS in an HIR model. Our findings elucidated that both preoperative and intraoperative therapy with DS lowered cellular damage and protected against toxic effects in liver HIR patients. These data imply that DS might be administered as a protective drug in elective and urgent liver surgical procedures against IRI (Tanrikulu et al. 2013). In both emergency and elective liver operations where likely ischemic periods are anticipated, DS may be given to safeguard the intestine against the detrimental effects of hepatic ischemia–reperfusion damage (Tanrikulu et al. 2011).

#### Radiation-induced hepatic injury

Radiotherapy is a key for the treatment, diagnosis, and staging of many diseases and cancers. Nevertheless, its detrimental effects on healthy tissues reduce its effectiveness. Ionizing radiation produces ions that hurt a variety of cell constituents, resulting in macromolecular alterations and biochemical abnormalities that lead to DNA strand breakage, protein oxidation, and loss of cellular integrity (Thariat et al. 2013). Ionizing radiation may generate a variety of biological consequences via multiple mechanisms, including inflammation, cancer formation, and even fatality. Radiation exposure from medical procedures, experimental work, nuclear power plant employment, nuclear warfare, or nuclear accidents may result in these effects (Nair et al. 2001). Based on research done by Mahgoub et al. on the radioprotective efficacy of DS, the administration of DS protects the liver and kidney of albino rats against harm caused by gamma radiation. Thus, DS may serve as a great radioprotective agent to safeguard rats against radiation-related injury (Mahgoub et al. 2020).

#### Heavy metals-induced hepatic intoxication

##### Cadmium (Cd)

The metallic element Cd is a member of the silver-white IIB class of elements. Its density is 8.6 g/mL, its melting point is 320.9 °C, and its boiling point is 765 °C. Its atomic number is 48. It is a naturally occurring inorganic salt that is frequently found in the environment and in the crust of the earth. The most often encountered Cd compounds are Cd acetate, Cd sulfide, Cd sulfoselenide, Cd selenium sulfide, Cd stearate, Cd oxide, Cd carbonate, Cd sulfate, and Cd chloride (Tchounwou et al. 2012; Zalups et al. 2003). Cd alters the balance of critical metals by interfering with their transport mechanisms and replacing them with oxygen-mediated ROS. This disturbance results in disruption of the antioxidant defense system, resulting in oxidative stress and LPO. Cd also depletes the stocks of critical metals and binds to thiol groups of GSH. Furthermore, it causes mitochondrial malfunction, intracellular Ca^2+^ imbalance, and abnormalities in DNA expression and ultimately induces apoptotic cell death (Kozlowski et al. 2014; Vickers 2017). Several studies have indicated the negative impacts of Cd on hepatocytes (Andjelkovic et al. 2019; Jurczuk et al. 2004; Karaca and Eraslan 2013; Koyu et al. 2006; Xu et al. 2017; Zhu et al. 2019).

When treating heavy metal-related lethargy, the recommended technique for therapy frequently includes the use of a chelator to bind and remove the metals that have entered the systemic circulation (Flora et al. 2008; Tandon et al. 2003; Xu et al. 1996). However, numerous antioxidant substances that have been employed to reduce hepatic oxidative stress have been studied within the scope of earlier investigations (Eşrefogˇlu et al. 2007; Layachi and Kechrid 2012; Vickers 2017; Zafeer et al. 2012). According to Ağır et al., DS has powerful antioxidant properties. Cd-induced liver damage is known to occur via oxidative stress and different mechanisms. In situations of probable Cd exposure, DS may be administered as a supplemental drug with main therapeutic approaches to minimize liver damage. Moreover, this flavonoid may be eaten with meals as a preventative approach (Ağır and Eraslan 2019).

##### Lead (Pb)

Pb is categorized as a heavy metal and has an atomic number of 82 (Jomova and Valko 2011; Rusyniak et al. 2010). Due to its numerous physical and chemical characteristics, Pb is used in different industrial areas (Gottesfeld et al. 2011). Inorganic Pb is a chemical that accumulates in the body when consumed via the lungs and gastrointestinal tract. However, absorption via the skin is rather modest, saving organic Pb. The respiratory system is the principal route of exposure for workers, with 4–50% of breathed Pb reaching the circulation. In regard to dietary consumption, approximately 10% of Pb is absorbed by the body. The particle size of Pb is critical for its inhalation absorption (Papanikolaou et al. 2005; Patrick 2006; Sakai 2000). In kids, the gastrointestinal transmission of Pb is greater than that in adults. The absorption and retention of Pb, as well as the architecture and makeup of the gastrointestinal system, might vary based on variables such as age, food, and environment. Exposure to Pb may impair zinc, iron, calcium, and phosphorus absorption from the gut (Alissa and Ferns 2011; Furst 2002; Papanikolaou et al. 2005; Ros and Mwanri 2003).

Pb exposure leads to several harmful processes, including the stimulation of oxidative stress, interactions with thiol groups, and interference with critical metals. Pb binds to thiol-containing enzymes, leading to an imbalance between oxidants and antioxidants. Pb suppresses the action of delta-aminolevulinic acid dehydratase and GSH reductase and influences the activities of GSH peroxidase and GSH-S-transferase (Ercal et al. 2001; Sanders et al. 2009; Wang et al. 2008). Pb exposure causes damage via the formation of ROS and the depletion of endogenous antioxidant reserves. This leads to an imbalance between ROS generation and antioxidant capability. In biological systems where ROS production is enhanced, the quantities of antioxidant reserves are concurrently lowered (Flora et al. 2012; Patrick 2006). Given that Pb is renowned for its hazardous effects, it is necessary to research alternative therapeutic approaches to mitigate its negative effects. Therefore, examining the possible impacts of DS, which has substantial antioxidant effects, is crucial. DS may show promise as a possible therapeutic drug to alleviate the detrimental consequences of Pb intoxication (Hajimahmoodi et al. 2014; Naso et al. 2016).

Mehmet et al. observed that DS was shown to be useful in mitigating the harm caused by Pb acetate. The deleterious impact of Pb acetate was reduced by the elimination of free radicals and the antioxidant properties of DS (Bozdağ and Eraslan 2020). As a result, DS may be utilized in important therapeutic processes as in cases of suspected lead acetate toxicity, both as a protective and supporting drug.

#### Aflatoxin (AF)-induced hepatotoxicity

AFs are mycotoxins generated by some *Aspergillus* strains, specifically *A. flavus* and *A. parasiticus*. Among these mycotoxins, AF B1 is well acknowledged as one of the most powerful natural carcinogens and is commonly found in a variety of food and feed items. Because of its carcinogenic qualities, it poses a substantial health risk and is a serious problem in food safety (Pitt 2000; Wu et al. 2009). CYP 1A2 and 3A4 are the major enzymes in the liver that convert AF B1 to AF B1-8,9-epoxide (AFBO) (Dohnal et al. 2014; Roze et al. 2013). AFB1 is bio-converted into four key metabolic processes, which include O-dealkylation, ketoreduction, epoxidation, and hydroxylation (Rawal et al. 2010).

CYP P450 produces AFBO, which causes mutagenesis activity. It combines with the guanine N7 site in DNA and RNA to form AF B1-N7-guanine (Aiko and Mehta 2015). However, the metabolites that interact with nucleic acids as well as other biological macromolecules may have carcinogenic implications (Larsson et al. 2003; Wild and Turner 2002; Wu et al. 2009). These deadly byproducts of AF serve as essential factors for detrimental consequences, which include suppression of RNA, DNA, and protein synthesis (Wang and Groopman 1999). ROS are formed from oxidative biotransformation, and it is crucial to analyze MDA, NO, and 4-hydroxynonenal (4-HNE) levels to determine the degree of cellular harm induced by free radicals. The antioxidant enzymatic capacity can also reflect the degree of LPO and the severity of oxidative stress (Jomova et al. 2023). Thus, oxidative stress is an efficient process for developing AF toxicity due to its modified pro-oxidant/antioxidant balance (Abdel-Hamid and Firgany Ael 2015; Eraslan et al. 2013; Ingawale et al. 2014; Karabacak et al. 2015).

Gökhan et al. (2017) reported the involvement of oxidative stress in AF-intoxicated animals. Oxidative stress and LPO were reduced by DS. This research verified that DS improved biochemical markers and histological results to levels similar to those of the control group with respect to oxidative stress markers, including NO, MDA, and 4-HNE levels, as well as antioxidant enzymes (Eraslan et al. 2017). The outcomes proved that DS could reduce the numerous unfavorable consequences of AF poisoning.

## Conclusion and future recommendations

**Fig. 2:**
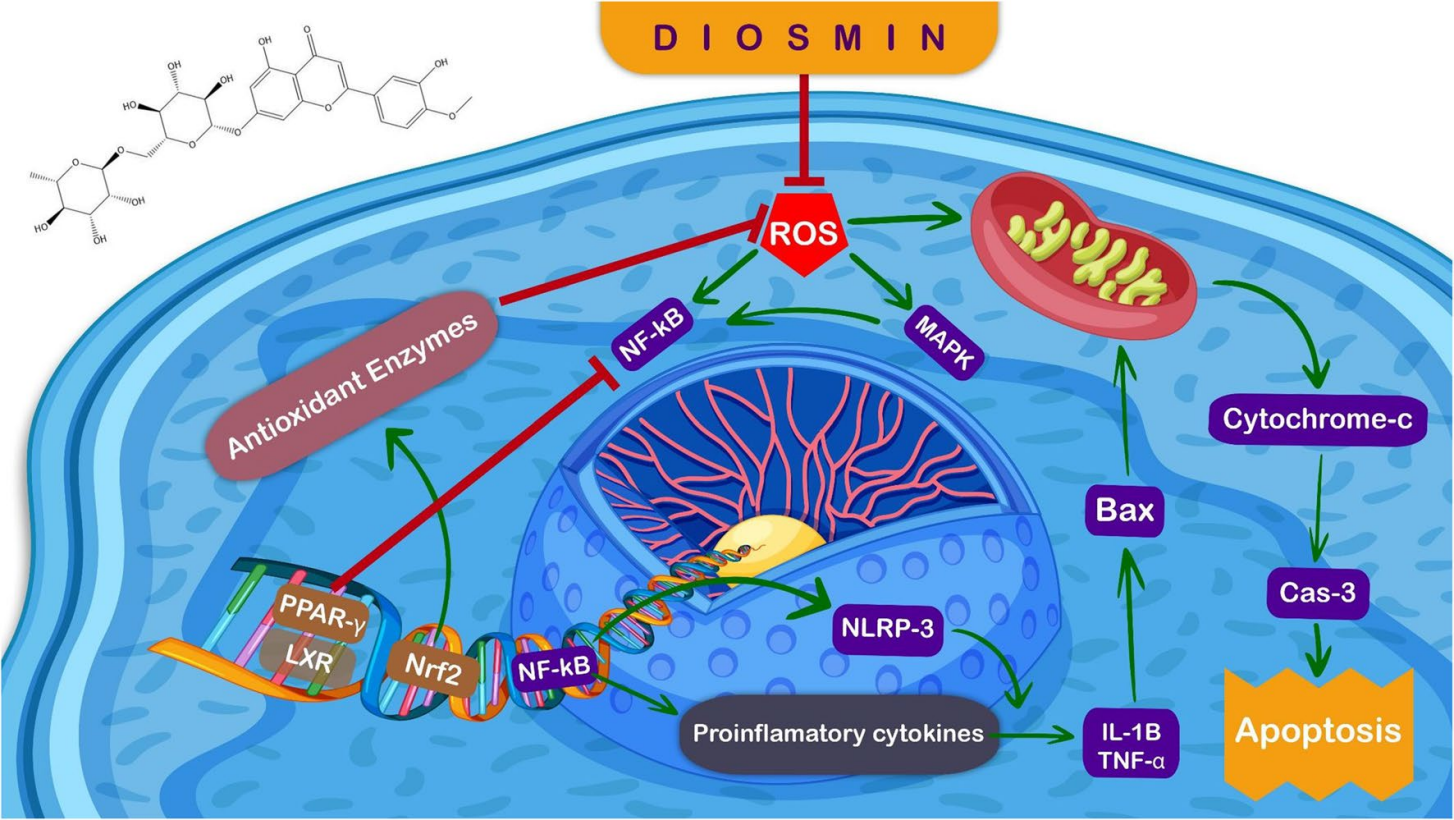
Underlying molecular mechanisms of DS. The underlying molecular mechanisms of DS are illustrated in this figure, along with the crosstalk between Nrf2, NF-κB, PPARγ, and MAPK signaling. DS modulates these signals and reduces oxidative stress and inflammatory response. PPARγ, MAPK, and NF-κB are examples of redox and inflammatory-sensitive signals that are triggered by increased ROS formation. ROS activates NF-κB. Genes that code for inflammation are transcriptionally activated when activated NF-κB translocates into the nucleus. Increased inflammatory gene transcription leads to increased levels of chemokines and cytokines such as TNF-α and IL-1β. These cytokines promote inflammation and cell death by increasing proapoptotic protein Bax expression. On the other hand, Nrf2 activation inhibits NF-κB. The active Nrf2 translocates to the nucleus and binds to ARE on DNA, activating the transcription of detoxifying antioxidant enzymes. These antioxidants prevent cell damage by inhibiting ROS and oxidative stress, which in turn prevents the production of inflammatory cytokines. Also, ROS generated exogenously or endogenously induce mitochondrial membrane depolarization and resulted cytochrome-C release and in turn activation of executioner protein of apoptosis Cas-3. Abbreviations: ROS, reactive oxygen species; TNF-α, tumor necrosis factor-alpha; IL-1β, interleukin-1β; PPARγ, peroxisome proliferator-activated receptor gamma; MAPK, mitogen-activated protein kinase; NF-κB, nuclear factor-kappa B; NLRP-3, NLR family pyrin domain containing-3; Nrf2, nuclear factor erythroid 2-related factor 2; Cas-3, caspase-3

Recently, DS has grabbed the attention of nutritionists, medical professionals, and researchers due to its promising preclinical applications in tackling various human ailments. This growing interest stems from the recognition of the beneficial properties of DS and its potential to address specific health conditions. This leads to an immense focus on exploring the potential avenues of DS in clinical settings and further investigating its efficacy in improving human health outcomes. Indeed, DS exhibits hepatoprotective effects through a variety of mechanisms, including eliminating ROS, suppressing oxidative stress, reducing the inflammatory response, and restoring normal hepatic function. By targeting these different pathways, DS has the potential to ameliorate hepatic damage and enhance hepatic health. Interestingly, A high safety profile characterizes DS. It also has hepatoprotective effects on various liver illnesses (summarized in Table 2), such as ALD, NAFLD, hepatic fibrosis, HIRI, HCC, and liver damage caused by radiation. Furthermore, DS has attractive hepatoprotective effects on environmental pollutants, including heavy metals. Mechanistically, DS exerts its hepatoprotective effects primarily through two mechanisms. First, it activates PPAR-γ and Nrf2, leading to antioxidant effects that help reduce oxidative stress. Second, DS suppresses NF-κB, NLRP3, and MAPK activities, as well as cytokine production (TNF-α and IL-1β), resulting in inflammation suppression. These anti-inflammatory effects can also be attributed to the activation of PPAR-γ, and Nrf2, which are NF-κB inhibitors (Fig. 2). These multifaceted actions contribute to the overall hepatoprotective properties of DS, making it an appealing choice for preventing and treating liver diseases.

**Table 2:** Summary of the preclinical hepatoprotective effects of DS

Hepatic disorders	Animal or cells or model	Main findings	References
Nonalcoholic fatty liver disease (NAFLD)	High-fat diet and streptozotocin-triggered NASH in rats	DS improved the histopathological NASH results DS minimized the levels of TNF-α, IL-6, and MDA DS boosted glucose and fat metabolism DS decreased liver TGF-β, α-SMA, and collagen content	(Gerges et al. 2020)
Alcoholic liver disease (ALD)	Ethanol-induced toxicity in rats	DS reduced the levels of the TNF-α, NF-kB, iNOS, and COX-2 well as the levels of signs of necrotic cell death DS has shown the potential to inhibit the generation of ethanol-induced ROS and increased antioxidants GSH, GPx and GR	(Tahir et al. 2013a, b)
Hepatic Fibrosis	Irradiation (IRR) exposed rats	DS therapy successfully suppressed pro-oxidants, stimulated antioxidants, relieved the liver inflammatory response DS repressed fibrosis-inducing cytokines, and boosted PPAR-γ levels DS therapy inhibited Wnt-β-catenin signaling caused via the IRR	(Hasan et al. 2017)
	Bile duct ligation (BDL)-induced fibrosis in rats	DS relieved fibrosis caused by bile duct ligation (BDL) DS attenuated oxidative damage, fibrosis progression, and abnormal hepatic functions DS downregulated NF-κB-p65, p38MAPK, Keap-1, and iNOS while downregulated Nrf-2, cytoglobin, and endothelial NOS levels	(Ali et al. 2018a, b)
Hepatocellular carcinoma (HCC)	DEN-induced HCC in Rats	DS reduced HCC induced by DEN DS stabilized fluctuating levels of enzymes, including AST, ALT, ALP, LDH, and xenobiotic enzymes DS in habited cell proliferation DS improves liver histopathology DS decreased the levels of TNF-α, NF-kB, iNOS, and COX-2	(Tahir et al. 2013a, b)
	HA22T human HCC cells both in vivo and in vitro in a mouse xenograft model	DS on HA22T human HCC cells both in vivo and in vitro in a mouse xenograft model. HA22T cells received different doses of DS and demonstrated considerable enhancement of apoptosis in the cells DS decreased tumor size in a xenograft model	(Dung et al. 2012)
Hepatic ischemia–reperfusion (HIR)	Hepatic ischemia–reperfusion (HIR) injury in rats	DS has shown the potential to inhibit the generation of ROS and increased antioxidants GSH, GPx, CAT and SOD, while it decreased MDA	(Tanrikulu et al. 2013)
Radiation-induced hepatic injury	Gamma-radiation-induced damage in rats	DS protects the liver from histological abrasions caused by gamma radiation DS increased antioxidants GSH and SOD, while it decreased MDA DS inhibited DNA damage	(Mahgoub et al. 2020)
Heavy metals-induced hepatic intoxication	Cd-induced liver damage in rats	DS has shown the potential to inhibit the generation of ROS and increased antioxidants GSH, GPx, CAT and SOD, while it decreased MDA and NO	(Ağır and Eraslan 2019)
	Pb-induced liver damage in rats	DS decreased the serum levels of LDH, AST, ALT, and ALP DS has shown the potential to inhibit the generation of ROS and increased antioxidants GSH, GPx, CAT and SOD, while it decreased MDA and NO	(Bozdağ and Eraslan 2020)
Aflatoxin (AF)-induced hepatotoxicity	Aflatoxin (AF)-induced hepatotoxicity in rats	DS improved biochemical markers and histological results DS increased the levels of GPx, CAT and SOD, while it decreased NO, MDA, and 4-HNE levels, as well as antioxidant enzymes	(Eraslan et al. 2017)

## Data Availability

No datasets were generated or analyzed during the current study.
